# The draft genome and pan-genome structure of *Paraclostridium bifermentans* strain T2 isolated from sheep faeces

**DOI:** 10.1016/j.dib.2023.109660

**Published:** 2023-10-10

**Authors:** Thuto Gomolemo Magome, Tsepo Ramatla, Prudent Mokgokong, Oriel Thekisoe, Kgaugelo Edward Lekota

**Affiliations:** Unit for Environmental Sciences and Management, North-West University, Potchefstroom, 2531, South Africa

**Keywords:** *Paraclostridium bifermentans*, Whole genome sequencing, Sheep faeces

## Abstract

*Paraclostridium bifermentans* is a Gram-positive, rod-shaped bacterium that can inhabit various mesophilic environments such as soil, marine habitats, and polluted waters. Some species of *Paraclostridium* are reported to cause fatal infections in humans, although mechanisms and capacity for adaptation are still unknown. We hereby present the whole genome sequence data of *P. bifermentans* T2 strain isolated from sheep faecal matter in Potchefstroom, South Africa. DNA libraries were sequenced on the Oxford Nanopore Mk1B platform. The generated sequence data was assembled and polished using Flye assembler. Genome data analysis yielded a genome size of 2 911,782 bp, comprising of a 27.8 % G + C content. Rapid Annotation using Subsystem Technology (RAST) showed that the draft genome of this strain consists of 6 514 coding sequences (CDS). The pan-genome was defined by a total of 16 288 CDSs, grouping the strain with the genome of *P. bifermentans* SampleS7P1. The draft genome sequence has been deposited in NCBI GenBank with the accession number of JAUPET000000000.

Specifications Table

**Table:** 

Subject	Microbial genomics
Specific subject area	Molecular microbiology and bioinformatics
Type of data	Table figures
How data were acquired	Whole genome sequencing was performed on the Oxford Nanopore Technology (ONT) MK1B sequencing platform
Data format	Raw and analysed
Parameters for data collection	Only culture collection of *P. bifermentans* strain used in this study is available at North West University.
Description of data collection	Pure culture isolate was used for genomic extractions and the genomic DNA was sequenced using Oxford Nanopore Technology MK1B. Whole genome identification of the strain was typed using Type Strain Genome Server (TYGS). The genome annotation was performed with Rapid Annotation using Subsystem Technology (RAST) and Prokka v.1.14.0. The pangenome analysis was carried out using Roary v3.6.8 and visualized on Phandango.
Data source location	Sheep faecal samples were collected in Matlwang village in Potchefstroom, South Africa
Data accessibility	➢ The 16S rRNA sequence obtained in this study have been deposited to the GenBank database with assigned accession number (OR545558) ➢ The genome sequence of *Paraclostridium bifermentans* T2 was deposited in NCBI GenBank under accession number JAUPET000000000.
Related research article	Ramatla, T., Khumalo, Z. T. H.,Matshotshi, A., Lekota, K. E., Taioe, M. O., & Thekisoe, O.(2023). Molecular detection of Coxiella burnetii and Coxiella species in rats and chickens from poultry farms in North West Province, South Africa. Veterinary Medicine and Science, 9, 2185–2191. https://doi.org/10.1002/vms3.1192

## 1 Value of the Data

• The data presented is imperative to understand the aetiology of *P. bifermentans* strains presented in this study.
• The genome sequence acts as a reference point for other researchers who want to understand the global evolution of *P. bifermentans*.
• The whole-genome sequence data of *P. bifermentans* strain T2 could be used in the development of further experiments for genotyping of *P. bifermentans* strains in other research projects.

## 2 Data Description

Here we report the whole genome sequencing data of *Paraclostridium bifementans* strain T2, together with its pangenome structure for taxonomic identification purpose. P*araclostridium bifermentans* strain T2 was isolated from sheep faeces in Matlwang village of Potchefstroom town, in the North West Province, South Africa. The genome sequencing was performed using the Oxford Nanopore Technology. The assembled genome was annotated using the rapid annotation with the RAST server (RAST) [1]. The genome contained 2 911 782 base pairs (bp) with a G + C content of 27.80 %. The genome includes 6 514 coding sequences and 23 RNAs. The assembly statistics and genomic features of P. *bifermentans* strain T2 are summarized in Table 1. The Genome Taxonomy Database (GTDB) [2] assigned strain T2 to *P. bifermentans* with an average nucleotide identity (ANI) of 96.52 % to the reference strain ATCC 683 (Table 2).

**Table 1:** De novo assembly statistics and genomic features of *Paraclostridium bifermentans* strain T2 based on RAST annotation.

Genome size	2 911 782
Number of contigs	11
GC content (%)	27.8
Shortest contig size	41 287
Median sequence size	101 593
Mean sequence size	264 707.5
Longest contig size	1 413 618
N50 value	385 420
L50 value	2
Number of Coding Sequences	6 514
Number of RNAs	23
Number of subsystems	218
NCBI Accession Number	JAUPET000000000

**Table 2:** Genome Taxonomy Database identification of the *Paraclostridium bifermentans* strain T2.

User Genome ID	FastANI Reference	FastANI taxonomy	FastANI ANI	Closest placement taxonomy
Strain T2	*Paraclostridium bifermentans* ATCC 683 (GCF_006802875.1)	d_Bacteria; p__*Firmicutes_A*; c__*Clostridia*; o__*Peptostreptococcales*; f__*Peptostreptococcaceae*; g__*Paraclostridium;* s__*Paraclostridium_bifermentans*	96.52	d_Bacteria; p__*Firmicutes*_A; c__*Clostridia;* o__*Peptostreptococcales*; f__*Peptostreptococcaceae*; g__*Paraclostridium*; s__*Paraclostridium bifermentans*

**Fig. 1:**
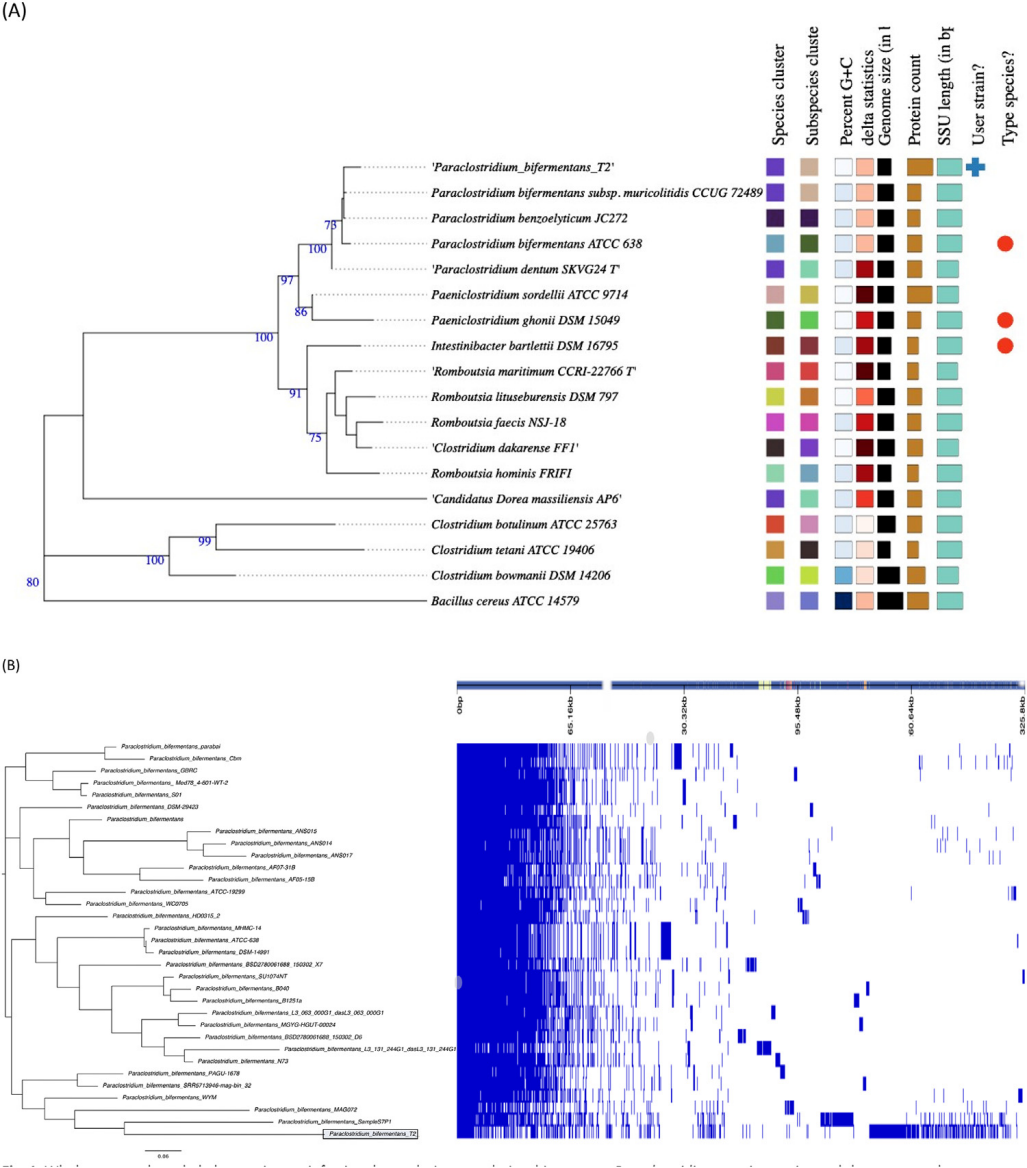
Whole genome based phylogenetic tree inferring the evolutionary relationships amongst *Paraclostridium* species strains and the sequenced draft genome strain T2. (A) Genome Blast Distance Phylogenies (GBDP) identified by TYGS [3] between *P. bifermentans* T2 and related genomes. (B) The pan-genome of the global *P. bifermentans* (*n* = 32) strains, placing the sequenced strain T2 highlighted in a box.

The whole-genome placement of Paraclostridium *bifermentans* T2 was used to determine the evolutionary relationship tree with other closely related to *Paraclostridium* species using the Type Strain Genome Server (TYGS) (https://tygs.dsmz.de) [3]. Fig. 1A shows that P. *bifermentans* T2 strain grouped in the *Paraclostridium* clade. A total of 16 288 genes made up the pan-genome, that consisted of 3 272 shell genes, 11 665 cloud genes, as well as 509 core genes and 842 soft core genes. The *P. bifermentans* strain T2 and *P. bifermentans* SampleS7P1 are closely related, as shown in Fig. 1B. The *P. bifermentans* strain T2 had putative proteins and genes such as polysaccharide type 8 biosynthesis and SPBc2 prophage-derived glycosyltransferase that are unique amongst the compared genomes used in this study.

## 3 Experimental Design, Materials, and Methods

### 3.1 Isolation

The *Paraclostridium bifermentans* strain was isolated from sheep faeces from Matlwang communal farming in the North West Province, South Africa. Isolation of *P. bifermentans* was carried out as described by Sasi Jyothsna et al. [4]. The identification of *P. bifermentans* isolates was performed by using 16S rRNA gene sequencing. Briefly, the bacteria were grown in tryptose sulfite cycloserine agar (Oxoid, UK) at 42°C for 6 h. After overnight incubation in reinforced clostridial medium (Oxoid, UK), colonies were pelleted. To ensure purity, the isolate was twice sub-cultured on Clostridia agar.

### 3.2 Genomic DNA extraction and sequencing

The bacterial genomic DNA was extracted using genomic DNA extraction kit (Invitrogen, USA) from pure cultures. Oxford Nanopore Technologies (ONT) sequencing was carried out on libraries prepared with ONT's rapid barcoding kit (catalogue number SQK-RBK004) using a MinION MK1B device with flow cell type R9.4.1 (catalogue number FLO-MIN106D). The isolate was sequenced at a 30X coverage, and Guppy v3.1.5 was used to base call, quality filter (minimum Q score, 10), demultiplex, barcode, as well as quality trimming of the sequenced reads.

### 3.3 Genome assembly, annotation, and data analysis

The generated FASTQ files were subjected to Nanoplot to assess the reads quality. Subsequently, raw data were processed and assembled using Flye (v2.9.2) [5]. The assembled genome was annotated using the rapid annotation with the RAST server (RAST) (rast.nmpdr.org) [2]. GTDB-Tk v1.6.0 [2] within the Kbase app [6] which incorporates the Fast Average Nucleotide Identity (ANI) was used to determine the identity and taxonomy of the T2 strain. *P. bifermentans* genome sequences (*n* = 32) were obtained from GenBank and further annotated together with the genome strain T2 from this study using Prokka v1.14.0 [7]. The annotated files were subsequently used for pan-genome analysis using Roary v. 3.6.8 [8]. The phylogenetic tree generated from Roary was visualized using Phandango (www.phandango.net) [3]. The genome sequence was typed using the Type Strain Genome Server (TYGS) https://tygs.dsmz.de, for a whole genome-based taxonomic analysis against other related bacterial genomes [3].

## Data Availability

The draft genome and pan-genome structure of Paraclostridium bifermentans strain T2 isolated from sheep faeces (Original data) (NCBI)

## References

[1] Aziz R.K., Bartels D., Best A.A., DeJongh M., Disz T., Edwards R.A., Formsma K., Gerdes S., Glass E.M., Kubal M., Meyer F. (2008). The RAST Server: rapid annotations using subsystems technology. BMC Genomics.

[2] Chaumeil P.A., Mussig A.J., Hugenholtz P., Parks D.H. (2020).

[3] Meier-Kolthoff J.P., Göker M. (2019). TYGS is an automated high-throughput platform for state-of-the-art genome-based taxonomy. Nat. Commun..

[4] Sasi Jyothsna T.S., Tushar L., Sasikala C., Ramana C.V. (2016). *Paraclostridium* benzoelyticum gen. nov., sp. nov., isolated from marine sediment and reclassification of *Clostridium bifermentans* as *Paraclostridium bifermentans* comb. nov. Proposal of a new genus Paeniclostridium gen. nov. to accommodate *Clostridium sordellii* and *Clostridium ghonii*. Int. J. Syst. Evol. Microbiol..

[5] Kolmogorov M., Yuan J., Lin Y., Pevzner P.A. (2019). Assembly of long, error-prone reads using repeat graphs. Nat. Biotechnol..

[6] Arkin A.P., Cottingham R.W., Henry C.S., Harris N.L., Stevens R.L., Maslov S., Dehal P., Ware D., Perez F., Canon S., Sneddon M.W. (2018). KBase: the United States department of energy systems biology knowledgebase. Nat. Biotechnol..

[7] Rai A., Ramana C.V., Uppada J., Sasikala C. (2015). Bergey's Manual of Systematics of Archaea and Bacteria.

[8] Seemann T. (2014). Prokka: rapid prokaryotic genome annotation. Bioinformatics.

